# Proliferation and estrogen signaling can distinguish patients at risk for early versus late relapse among estrogen receptor positive breast cancers

**DOI:** 10.1186/bcr3481

**Published:** 2013-09-23

**Authors:** Giampaolo Bianchini, Lajos Pusztai, Thomas Karn, Takayuki Iwamoto, Achim Rody, Catherine M Kelly, Volkmar Müller, Marcus Schmidt, Yuan Qi, Uwe Holtrich, Sven Becker, Libero Santarpia, Angelica Fasolo, Gianluca Del Conte, Milvia Zambetti, Christos Sotiriou, Benjamin Haibe-Kains, W Fraser Symmans, Luca Gianni

**Affiliations:** 1 San Raffaele - Scientific Institute, Milan, Italy; 2 Yale Cancer Center, New Haven, CT, USA; 3 J.W. Goethe University, Frankfurt, Germany; 4 University Graduate School of Medicine and Dentistry, Okayama, Japan; 5 University Medical Center Schleswig-Holstein, Campus Lübeck, Lübeck, Germany; 6 Mater Misericordiae University Hospital, Dublin, Ireland; 7 University Hospital Hamburg-Eppendorf, Hamburg, Germany; 8 Gutenberg-University, Mainz, Germany; 9 The University of Texas M. D. Anderson Cancer Center, Houston, TX, USA; 10 Translational Research Unit and Istituto Toscano Tumori, Prato, Italy; 11 Institut Jules Bordet, Univerité Libre de Bruxelies, Brussels, Belgium; 12 Bioinformatics and Computational Genomics Laboratory, Institut de recherches cliniques de Montréal, Montreal, Canada

## Abstract

**Introduction:**

We examined if a combination of proliferation markers and estrogen receptor (ER) activity could predict early versus late relapses in ER-positive breast cancer and inform the choice and length of adjuvant endocrine therapy.

**Methods:**

Baseline affymetrix gene-expression profiles from ER-positive patients who received no systemic therapy (n = 559), adjuvant tamoxifen for 5 years (cohort-1: n = 683, cohort-2: n = 282) and from 58 patients treated with neoadjuvant letrozole for 3 months (gene-expression available at baseline, 14 and 90 days) were analyzed. A proliferation score based on the expression of mitotic kinases (MKS) and an ER-related score (ERS) adopted from Onco*type* DX^®^ were calculated. The same analysis was performed using the Genomic Grade Index as proliferation marker and the luminal gene score from the PAM50 classifier as measure of estrogen-related genes. Median values were used to define low and high marker groups and four combinations were created. Relapses were grouped into time cohorts of 0–2.5, 0–5, 5-10 years.

**Results:**

In the overall 10 years period, the proportional hazards assumption was violated for several biomarker groups indicating time-dependent effects. In tamoxifen-treated patients Low-MKS/Low-ERS cancers had continuously increasing risk of relapse that was higher after 5 years than Low-MKS/High-ERS cancers [0 to 10 year, HR 3.36; p = 0.013]. High-MKS/High-ERS cancers had low risk of early relapse [0–2.5 years HR 0.13; p = 0.0006], but high risk of late relapse which was higher than in the High-MKS/Low-ERS group [after 5 years HR 3.86; p = 0.007]. The High-MKS/Low-ERS subset had most of the early relapses [0 to 2.5 years, HR 6.53; p < 0.0001] especially in node negative tumors and showed minimal response to neoadjuvant letrozole. These findings were qualitatively confirmed in a smaller independent cohort of tamoxifen-treated patients. Using different biomarkers provided similar results.

**Conclusions:**

Early relapses are highest in highly proliferative/low-ERS cancers, in particular in node negative tumors. Relapses occurring after 5 years of adjuvant tamoxifen are highest among the highly-proliferative/high-ERS tumors although their risk of recurrence is modest in the first 5 years on tamoxifen. These tumors could be the best candidates for extended endocrine therapy.

## Introduction

A sizable risk of late recurrence exists in estrogen receptor (ER)-positive breast cancers after completion of 5 years of adjuvant tamoxifen therapy [1,2]. This justifies consideration of extended endocrine treatment in ER-positive early-stage cancers. Letrozole after 5 years of tamoxifen was found to reduce recurrence and improve overall survival [1], demonstrating that some micrometastatic foci retain sensitivity to endocrine therapy even after 5 years of tamoxifen. Continuing tamoxifen to 10 years has also demonstrated an overall survival benefit compared to 5 years of tamoxifen [2]. However, the small absolute benefit should be weighted against the potential side effects of prolonged endocrine therapy [3]. Available molecular markers do not identify patients who are at high risk for late recurrence (after 5 yrs) who would be the best candidates for extended endocrine therapy. While the assays of Recurrence Score and PAM50 predict prognosis for over 10 years, they are most prognostic in the first 5 years and have limited predictive values for late relapses [4,5]. Both tests rely on measuring genes associated with proliferation and estrogen receptor signaling [4,5]. However, the combination of these markers in these tests is fixed and does not allow detection of time-varying effect or interactions between markers. Given the independent prognostic and predictive values of proliferation and estrogen signaling, we examined in a time-dependent way whether a combination of proliferation, measured by the Mitotic Kinase Gene Expression Score (MKS), and ER-related gene expression, measured by an estrogen-related gene expression score (ERS), could improve the ability of these variables to predict early versus late relapses in women with ER-positive breast cancer. This information could help in selecting patients for extended adjuvant endocrine therapy based on their persistent risk for late relapses.

## Methods

### Tumor samples

Publicly available Affymetrix gene expression data from 683 patients (cohort 1; GSE6532 [6] GSE9195 [7] GSE17705 [8] and GSE12093 [9]) with ER-positive breast cancers treated with 5 years adjuvant tamoxifen and 559 ER-positive, untreated, node-negative breast cancers (GSE2034 [10] GSE7390 [11] GSE11121 [12] GSE5327 [13] GSE2990 [14] and GSE6532 [6]) were analyzed. Duplicated patients were removed from the different datasets. We also assessed an independent series of ER-positive patients treated with adjuvant tamoxifen for 5 years (cohort 2, n = 282, GSE26971 [15]) and 58 ER-positive patients treated with neoadjuvant letrozole for 3 months for which gene expression profiles are available at baseline, 14 and 90 days after beginning of the neoadjuvant treatment (GSE20181 [16]). In this series, patients with a volume reduction higher than 50% over the 3-month treatment period were considered as clinical responders.

Although of limited sample size, cohort 2 was added to our analysis to provide a qualitative confirmation of our findings. Multivariate analysis of this series was not feasible due to the small number of late events (n = 18). Gene expression data used in this study were publicly available and ethical approvals have been obtained as reported in the original publications to which the datasets referred.

### Description of molecular marker used for analysis

The proliferation score was calculated as the average expression of 12 mitotic kinases to produce the MKS as previously described [17]. The ERS was defined as the average expression of the four genes from the ER-associated group of the Onco*type* DX test (Additional file 1: Table S1) [18]. None of these gene scores was developed or optimized on the current data and therefore our results represent an independent validation of their predictive and prognostic values. To avoid over-fitting of an arbitrary marker threshold to the data, median values were calculated over the combined series of untreated patients and cohort 1 of the tamoxifen-treated patients, and used to define high (that is, above the median) and low (that is, below the median) expression groups. A similar approach was used in cohort 2. By combining these two metrics, four biomarker groups were created (low-MKS/high-ERS; low-MKS/low-ERS; high-MKS/high-ERS and high-MKS/low-ERS).

In order to assess the robustness of our findings, we also tested other measures of proliferation and ER signaling (Additional file 1: Table S1) [19]. We assessed the ability of four previously reported gene signatures to predict early versus late relapse including the PAM50 subtypes [19], the GGI (Genomic Grade Index) [14], the MammaPrint [20] and the three-gene classifier (ER, human epidermal growth factor receptor 2 (HER2) and Aurora Kinase A (AURKA) [21] (Additional file 2: Supplementary Methods).

### Statistical analysis

Statistical analyses were performed using BRB-ArrayTools v4.1.0 - Beta_3 Release [22] and R software v2.9.0. All statistical tests were two-sided. Distant event-free survival was the primary endpoint. For each biomarker, the proportional hazards assumption for Cox regression model was assessed based on the scaled Schoenfeld residuals.

In order to assess the time-dependent prognostic value of our biomarkers groups, we examined recurrences rates in three distinct time intervals, including 0.0 to 2.5 years, 0.0 to 5.0 years and 5.0 to 10.0 years. These intervals were selected because they correspond to clinically important decision points. Patients at high risk for relapse after 5 years of tamoxifen (that is, late relapse) may be the optimal candidates for extended adjuvant endocrine therapy. The period from 0.0 to 2.5 years before the conventional switch to aromatase inhibitors is when the selection of the upfront endocrine treatment (tamoxifen versus aromatase inhibitors) is made. Landmark analysis of the Kaplan-Meier estimates by biomarker groups in different time intervals was used. The estimates of the hazard function by biomarker groups were performed using the 'muhaz’ R package (v 1.2.5) [23]. The univariate and multivariate hazard ratios were estimated using Cox regression analysis by the Wald test.

## Results

### Patient characteristics and correlation between clinical and molecular variables

DEFS information was available for 673 of the 683 patients who received adjuvant tamoxifen in cohort 1. The median follow up was 9.15 years. DEFS information was available for 553 of the 559 patients who did not receive systemic adjuvant therapy and their median follow up was 9.9 years. The treated (cohort 1) and untreated patient cohorts were significantly different in age, tumor (T) size, nodal status and histological grade (Table 1).

**Table 1:** Summary of patient and tumor characteristics

	**Tamoxifen cohort 1**		**Untreated**			**Low MKS**				**High MKS**					**Tamoxifen**	
						**High ERS**		**Low ERS**		**High ERS**		**Low ERS**			**cohort 2**	
**Characteristic**	**N**	**%**	**N**	**%**	***P***^**a**^	**N**	**%**	**N**	**%**	**N**	**%**	**N**	**%**	***P***^**b**^	**N**	**%**
**Patients**	683		559			373		248		248		373			282	
**Age, years**																
≤50	43	6.3	240	42.9	**<0.0001**	82	22.0	47	19.0	57	23.0	96	25.7	0.247	8	2.8
>50	498	72.9	306	54.8		240	64.3	172	69.3	160	64.5	233	62.5		176	62.4
Unknown	142	20.8	13	2.3		51	13.7	29	11.7	31	12.5	44	11.8		98	34.8
**Tumor (T) stage**																
T1	214	31.3	199	35.6	**<0.0001**	145	38.9	102	41.1	74	29.8	92	24.7	**0.003**	109	38.7
T2/T3	293	42.9	140	25.0		126	33.8	82	33.1	84	33.9	141	37.8		168	59.5
Unknown	176	25.8	220	39.4		102	27.3	64	25.8	90	36.3	140	37.5		5	1.8
**Nodal status**																
Negative	403	59.0	559	100.0	**<0.0001**	295	79.1	192	77.4	191	77.0	284	76.1	0.962	147	52.1
Positive	258	37.8	0	0.0		76	20.4	50	20.2	52	21.0	80	21.5		111	39.4
Unknown	22	3.2	0	0.0		2	0.5	6	2.4	5	2.0	9	2.4		24	8.5
**Grade**																
1	108	15.8	66	11.8	**0.0002**	86	23.1	54	21.8	18	7.3	16	4.3	**<0.0001**	26	9.2
2	258	37.8	242	43.3		159	42.6	105	42.3	108	43.5	128	34.3		114	40.4
3	111	16.3	152	27.2		29	7.8	32	12.9	63	25.4	139	37.3		44	15.6
Unknown	206	30.2	99	17.7		99	26.5	57	23.0	59	23.8	90	24.1		98	34.8
**HER2 by gene**^**c**^																
HER2-positive	27	4.0	44	7.9	0.169	1	0.3	8	3.2	7	2.8	55	14.8	**<0.0001**	-	0.0
HER2-negative	647	94.7	515	92.1		371	99.4	239	96.4	239	96.4	313	83.9		-	0.0
Unknown	9	1.3	0	0.0		1	0.3	1	0.4	2	0.8	5	1.3		282	100.0
**Distant events, time cohorts**																
0 to 10 yrs	137		144		-	42		37		65		137		**-**	64	
0 to 2.5 yrs	46		55			8		9		17		67			26	
2.5 to 5 yrs	48		55			19		10		21		53			20	
>5 yrs	43		34			15		19		26		17			18	
Unknown	10		6			2		6		3		5			-	

^a^Only patients with known clinical information were considered for comparison between tamoxifen-treated (cohort 1) and untreated patients. ^b^Only patients with known clinical information were considered for comparison among the four molecular groups. *P-*values in boldface were statistically significant at *P* <0.05. These molecular groups referred to the tamoxifen-treated (cohort 1) and untreated patients. ^c^HER2 status was defined according to Haibe-Kains *et al*. [21]. N, number of patients; MKS, mitotic kinase score; ERS, estrogen-related score; HER2: human epidermal growth factor receptor 2.

There was no association between the four molecular groups (low-MKS/high-ERS, low-MKS/low-ERS, high-MKS/high-ERS and high-MKS/low-ERS), nodal status or age. However, high-MKS cancers had higher grade and larger T size (T2/T3). The majority of HER2-positive tumors (77.5%) were in the high-MKS/low-ERS group.

### Time-varying prognostic values of clinical and molecular variables in adjuvant tamoxifen-treated patients

We assessed the prognostic values of clinical and molecular variables for 10 years DEFS using Cox analysis in tamoxifen-treated patients (cohort 1) and tested the assumption of proportional hazards for each model (Table 2). Age, T size and nodal status did not violate the proportional hazards assumption but some molecular variables showed violation of the proportional hazard assumption indicating that their prognostic values varied over time.

**Table 2:** Univariate Cox analysis of clinical variables and molecular markers for tamoxifen-treated patients (cohort-1)

**Variable**	**0 to 10 years DEFS**			**0 to 5 years DEFS**			**5 to 10 years DEFS**		
	**Hazard ratio (95% CI)**	***P***^**a**^	***P***^**b**^	**Hazard ratio (95% CI)**	***P***^**a**^	***P***^**b**^	**Hazard ratio (95% CI)**	***P***^**a**^	***P***^**b**^
**Age**	1.02 (1.00, 1.04)	**0.021**	0.657	1.02 (0.99, 1.04)	0.153	0.214	1.04 (1.00, 1.08)	**0.042**	0.168
**Stage T2/3 vs T1**	2.71 (1.76, 4.19)	**<0.0001**	0.581	2.51 (1.47, 4.26)	**0.0007**	0.211	3.14 (1.49, 6.64)	**0.003**	0.164
**Node-positive vs -negative**	2.47 (1.75, 3.48)	**<0.0001**	0.964	2.51 (1.65, 3.82)	**<0.0001**	0.732	2.67 (1.44, 4.97)	**0.002**	0.941
**Grade**									
Intermediate vs low	2.19 (1.20, 3.99)	**0.011**	0.11	3.97 (1.57, 9.99)	**0.003**	0.175	1.04 (0.44, 2.45)	0.934	0.192
High vs low	2.96 (1.56, 5.62)	**0.0009**		5.05 (1.92, 13.3)	**0.001**		1.66 (0.67, 4.12)	0.277	
**HER2-positive vs -negative**	2.64 (1.43, 4.90)	**0.002**	**0.029**	3.68 (1.91, 7.10)	**0.0001**	0.369	0.72 (0.11, 5.26)	0.75	0.141
**High MKS vs low MKS**	2.96 (2.07, 4.25)	**<0.0001**	**0.04**	3.89 (2.42, 6.24)	**<0.0001**	0.473	1.90 (1.04, 3.46)	**0.036**	0.377
**Low ERS vs high ERS**	2.00 (1.41, 2.83)	**<0.0001**	**0.0008**	3.26 (2.04, 5.19)	**<0.0001**	0.091	0.91 (0.50, 1.66)	0.763	0.728
**MKS/ERS combined groups**									
LowMKS/lowERS vs lowMKS/highERS	2.37 (1.29, 4.35)	**0.005**	**<0.0001**	1.98 (0.87, 4.51)	0.104	0.285	3.36 (1.29, 8.73)	**0.013**	0.862
HighMKS/highERS vs lowMKS/highERS	3.66 (2.04, 6.57)	**<0.0001**		2.46 (1.09, 5.54)	**0.029**		6.59 (2.62, 16.6)	**<0.0001**	
HighMKS/lowERS vs lowMKS/highERS	5.35 (3.12, 9.17)	**<0.0001**		7.78 (3.97, 15.3)	**<0.0001**		1.46 (0.45, 4.79)	0.531	
**Other genomic signatures**									
**High GGI vs low GGI**	2.73 (1.94, 3.86)	**<0.0001**	**0.005**	3.81 (2.43, 5.97)	**<0.0001**	0.296	1.64 (0.90, 2.99)	0.109	**0.02**
**Mammaprint poor vs good**	2.93 (1.91, 4.49)	**<0.0001**	0.22	3.59 (2.02, 6.30)	**<0.0001**	0.768	2.30 (1.16, 4.56)	**0.017**	0.184
**Three-gene model SCMGENE**									
ER+/HER2-highP vs ER+/HER2-lowP	2.13 (1.47, 3.08)	**<0.0001**	**0.017**	2.58 (1.59, 4.18)	**0.0001**	0.585	1.60 (0.87, 2.95)	0.127	0.197
HER2+ vs ER+/HER2-lowP	4.06 (2.09, 7.86)	**<0.0001**		6.63 (3.17, 13.9)	**<0.0001**		0.89 (0.12, 6.64)	0.909	
ER-/HER2- vs ER+/HER2-lowP	2.55 (1.01, 6.42)	**0.048**		4.70 (1.79, 12.3)	**0.002**		NA		
**PAM50**									
LumB vs lumA	3.01 (2.01, 4.51)	**<0.0001**	**0.004**	3.84 (2.21, 6.65)	**<0.0001**	0.109	2.26 (1.20, 4.24)	**0.011**	0.147
HER2 vs lumA	8.88 (4.36, 18.1)	**<0.0001**		14.4 (6.37, 32.8)	**<0.0001**		2.37 (0.31, 17.9)	0.403	
Basal vs lumA	3.03 (1.07, 8.58)	**0.036**		6.48 (2.17, 19.4)	**0.0008**		NA		

^a^*P-*value generated by univariate Cox analysis. ^b^*P-*value for the test of the proportional hazard assumption based on the scaled Schoenfeld residuals. P-values in boldface were statistically significant at *P*<0.05. DEFS, distant event-free survival; MKS, mitotic kinase score; ERS, estrogen-related score; GGI, genomic grade index; Lum, luminal; ER+/HER2-lowP, ER-positive/human epidermal growth factor receptor 2 (HER2)-negative low proliferation; ER+/HER2-highP, ER-positive/HER2-negative high proliferation; NA, not applicable.

**Figure 1:**
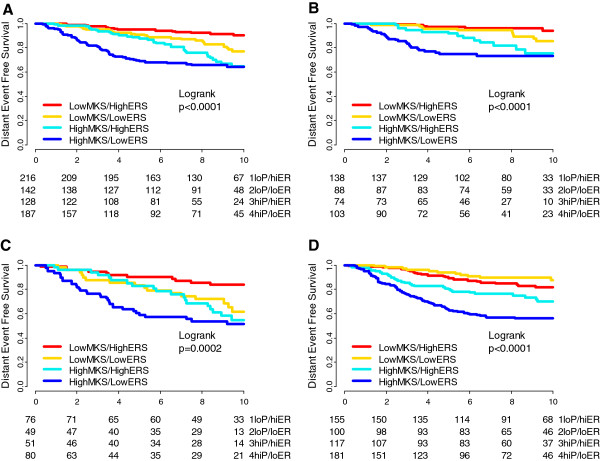
**Kaplan-Meier estimates of distant event-free survival (DEFS) by marker groups in tamoxifen-treated and untreated patients.** Kaplan-Meier estimates of 10-year DEFS of the four biomarker groups obtained by combining low and high MKS (mitosis kinome score) and ERS (estrogen-related score). Results are plotted separately for tamoxifen-treated (all patients) **(A)**, tamoxifen-treated (node negative) **(B)**, tamoxifen-treated (node positive) **(C)**, and tamoxifen-untreated patients **(D)**. loP, low MKS; hiP, high MKS; loER, low ERS; hiER, high ERS.

We divided the time axis into less than 5 and 5 to 10 years and estimated the hazard ratios (HRs) separately for each variable in both time periods. High-proliferation tumors had a higher risk of relapse during the entire period, but the risk decreased significantly over time. HER2-positive tumors had a higher risk of relapse only during the first 5 years. The ERS also had a strong time dependent effect (*P* = 0.0008 over 10 years). The HRs for recurrence of low- versus high-ERS groups in the first 2.5 years, from 2.5 to 5.0 years, and >5.0 years were 8.36 (95% CI 3.37, 21.7), 1.93 (95% CI 1.07, 3.48) and 0.91 (95% CI 0.50, 1.66), respectively. The four-group model obtained by combining MKS and ERS had the strongest time-dependent effect (*P* <0.0001). Kaplan-Meier analysis of the four biomarker groups is represented in Figure 1 for the tamoxifen-treated patients (Figure 1A). The time-dependent effect of biomarkers was qualitatively similar in node-negative (Figure 1B) and -positive (Figure 1C) tumors.

### Time-varying prognostic values of clinical and molecular variables in untreated patients

We performed a similar analysis in patients who received no adjuvant therapy (Additional file 3: Table S2). Age and T size did not violate the proportional hazards assumption over 10 years. The low-ERS group had a constant, slightly higher risk of recurrence than the high-ERS group in the first 5 years (HR 1.90, 95% CI 1.29, 2.80; *P* = 0.001) without a significant violation of the proportional hazard assumption (*P* = 0.127). High-proliferation tumors (that is, high histological grade, high-MKS, high-GGI, luminal B) had a higher risk of relapse over the entire 10-year period, but the risk decreased significantly after the first 5 years. There was a trend for a higher relative rate of relapse in low proliferation tumors after 5 years (Additional file 4: Figure S1). In the absence of endocrine therapy, the four-group model had only a trend for time-dependent effect (*P* = 0.065) (Figure 1D).

### Prediction of early and late relapse in tamoxifen-treated patients

**Figure 2:**
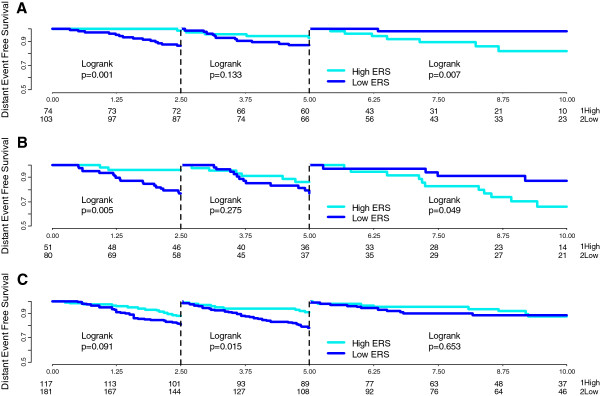
**Time-dependent assessment of estrogen-related score (ERS) in the high mitosis kinase score (MKS)-group in both tamoxifen-treated and untreated patients.** Landmark analyses of the Kaplan-Meier estimates of distant event-free survival (DEFS) for highly proliferative tumors (high-MKS) according to ERS groups (high and low) during the first 2.5 years (left side of each panel), from 2.5 to 5 years (center of each panel) and from 5 to 10 years (right side of each panel) are shown. Results are plotted separately for tamoxifen-treated, node-negative **(A)**, tamoxifen-treated, node-positive **(B)** and tamoxifen-untreated, node-negative **(C)** patients.

Among the tamoxifen-treated patients, cancers with low proliferation and low ERS were associated with a continuously increasing risk of relapse over 10 years (Table 2), that was higher between 5 to 10 years than in 0 to 5 years (HR 3.36, 95% CI 1.29, 8.73; *P* = 0.013) (Additional file 4: Figure S2A). Low proliferation and high-ERS tumors had an excellent prognosis at 10 years, even after stopping tamoxifen at 5 years (Figure 1). In the high-proliferation group, ERS had an even stronger time-dependent prognostic value (*P* <0.0001). Among high proliferation cancers, the HRs for the low- versus high-ERS groups in the time periods 0.0 to 2.5, 2.5 to 5.0, and >5.0 to 10.0 years were 11.8, 95% CI 2.83, 49.3; *P* = 0.0007, 1.80, 95% CI 0.88, 3.69; *P* = 0.110, and 0.23, 95% CI 0.08, 0.61; *P* = 0.003), respectively indicating very high risk of early relapse among high-proliferation low-ESR cancers (Additional file 4: Figure S2A). This was similar in node-negative (Figure 2A) and -positive (Figure 2B) patients. To test if this effect was independent of the threshold used, we also examined the time dependence when ESR was assessed by tertiles or as continuous variables. The lowest tertile of ERS compared to the highest showed a higher risk of early relapse within 0 to 5 years (HR 3.93, 95% CI 1.99, 7.75; *P* <0.0001) and a lower risk of late relapse between 5 to 10 years (HR 0.09, 95% CI 0.01, 0.70; *P* = 0.021) (Additional file 4: Figure S3). ERS analyzed as a continuous variable produced similar results, indicating a robust finding.

These findings also indicate that the prognostic value of ER-associated genes for late recurrence is different in low- and high-proliferation cancer groups (Additional file 4: Figure S2A). In the 5- to 10-year interval, Cox analysis including MKS and ERS as continuous variables and their interaction term showed that they were each independently significant (*P* = 0.035 and *P* = 0.048, respectively). The interaction term was also significant (*P* = 0.027). In the 0.0- to 2.5-year interval, almost all recurrences occurred in the high-MKS/low-ERS group (HR 6.53, 95% CI 3.48, 12.2; *P* <0.0001) compared to other groups (Additional file 4: Figure S4B). In this high-risk group the nodal status was not significantly prognostic (*P* = 0.098) (Additional file 4: Figure S4F), but in the other molecular groups enriched for more endocrine-sensitive tumors, almost all relapses were observed in the node-positive cohort (HR 6.56, 95% CI 1.83, 23.5; *P* = 0.0008) (Additional file 4: Figure S4E). The average ERS of high-MKS tumors that relapsed at 0.0 to 2.5, 2.5 to 5.0 and >5.0 to 10.0 years was progressively and significantly higher in late relapses (Additional file 4: Figure S5B).

Results of a Cox multivariate analysis for the 0.0- to 2.5- and 5.0- to 10.0-year time periods are presented in Table 3. After adjustment for clinical variables, high-MKS/high-ERS (HR 4.59, 95% CI 1.56, 13.5; *P* = 0.006) and low-MKS/low-ERS groups (HR 3.53, 95% CI 1.21, 10.2; *P* = 0.021) remained at higher risk of late relapses than the low-MKS/high-ERS group.

**Table 3:** Multivariate Cox analysis of clinical variables and marker groups in tamoxifen-treated (cohort 1) and untreated patients

	**Tamoxifen-treated patients (cohort 1)**				**Untreated patients**			
	**0.0 to 2.5 years**^**a**^		**5.0 to 10.0 years**^**a,c**^		**0.0 to 2.5 years**^**b**^		**5.0 to 10.0 years**^**b,c**^	
**Variable**	**Hazard ratio (95% CI)**	***P***	**Hazard ratio (95% CI)**	***P***	**Hazard ratio(95% CI)**	***P***	**Hazard ratio(95% CI)**	***P***
Age, analyzed as a continuous variable	1.04 (0.99, 1.08)	0.071	1.02 (0.98, 1.07)	0.279	1.01 (0.98, 1.05)	0.357	1.04 (0.99, 1.08)	0.063
Tumor (T) stage, T2/3 vs T1	2.29 (0.87, 6.08)	0.094	2.90 (1.22, 6.91)	**0.016**	1.88 (0.74, 4.80)	0.183	1.22 (0.48, 3.12)	0.677
Nodal status, positive vs negative	2.92 (1.23, 6.88)	**0.014**	2.46 (1.15, 5.23)	**0.021**	-	-		
Grade 2 or 3 vs 1	1.51 (0.33, 6.78)	0.592	0.72 (0.31, 1.69)	0.451	2.13 (0.28, 16.4)	0.467	1.07 (0.29, 3.95)	0.923
HER2-positive vs -negative	1.42 (0.33, 6.78)	0.528	1.63 (0.31, 1.69)	0.667	4.24 (1.36, 13.1)	**0.013**	2.89 (0.57, 14.6)	0.198
MKS/ERS (ref lowMKS/highERS)								
lowMKS/lowERS	2.65 (0.48, 14.7)	0.269	3.53 (1.21, 10.2)	**0.021**	1.67 (0.10, 27.1)	0.717	0.46 (0.09, 2.33)	0.349
highMKS/highERS	1.28 (0.18, 9.23)	0.807	4.59 (1.56, 13.5)	**0.006**	11.6 (1.41, 95.2)	**0.023**	1.82 (0.53, 6.27)	0.343
highMKS/lowERS	10.0 (2.21, 45.5)	**0.003**	0.95 (0.20, 4.70)	0.947	9.56 (1.17, 77.8)	**0.035**	1.51 (0.44, 5.14)	0.508

Note: only patients with all data available were considered in multivariate analysis. ^a^Time interval 0.0 to 2.5 years: 434 patients (29 events); time interval 5.0 to 10.0 years: 323 patients (34 events). ^b^Time interval 0.0 to 2.5 years: 320 patients (20 events); time interval 5.0 to 10.0 years: 256 patients (19 events). Cox multivariate analysis was performed also excluding tumor stage (which accounted for the majority of the missing data) with similar results (458 patients (40 events) and 349 patients (28 events) in 0.0 to 2.5 and 5.0 to 10 years, respectively). ^c^*P* = 0.0002 (tamoxifen, 0.0 to 2.5 years), *P* = 0.003 (tamoxifen, 5.0 to 10.0 years), *P* = 0.006 (untreated, 0.0 to 2.5 years), *P* = 0.361 (untreated, 5.0 to 10.0 years) for the comparison of the multivariate models with and without the MKS/ERS groups (by the likelihood-ratio test). MKS, mitotic kinase score; ERS, estrogen-related score. *P-*values in boldface were statistically significant at *P* <0.05.

### Prediction of early and late relapse in untreated patients

ERS was not predictive of early versus late relapse when proliferation (MKS) was low (Additional file 3: Table S2). In the high-MKS group, there was a constantly higher risk of relapse for the low-ERS group over 10 years (HR 1.80, 95% CI 1.18, 2.73); *P* = 0.006) (Figure 2C and Additional file 3: Table S2). In the first 2.5 years, the high-MKS/low-ERS group had a higher risk of relapse than the other groups combined (Additional file 4: Figure S4A) and the HER2-positive patients were also at significantly higher risk of relapse. Cox multivariate analysis showed that none of the variables was prognostic for late relapse (Table 3). When the average ERS was compared between tumors relapsing at 0.0- to 2.5-, 2.5- to 5.0- and 5.0- to 10-year time intervals we did not observe any significant difference by proliferation group (Additional file 4: Figure S5C and S5D).

### A combination of proliferation and ER-related genes can further risk-stratify patients for early and late relapse within conventional prognostic risk categories assigned by PAM50 and Mammaprint

To test the robustness of our observations, we performed the same analysis substituting the genomic grade index for MKS as a proliferation marker and the luminal gene score from the PAM50 classifier for the ERS as a measure of estrogen-related genes. Results using the combination of these markers also showed higher risk of early relapse in highly proliferative, low estrogen-score cancers and substantial risk for late relapse in highly proliferative and high estrogen-score tumors (Additional file 4: Figure S6). We also noted that ESR1 mRNA expression levels did not correlate strongly with either the ERS or luminal gene scores, an indication that these scores capture molecular information not provided by ESR1 expression levels alone (Additional file 4: Figure S7).

**Figure 3:**
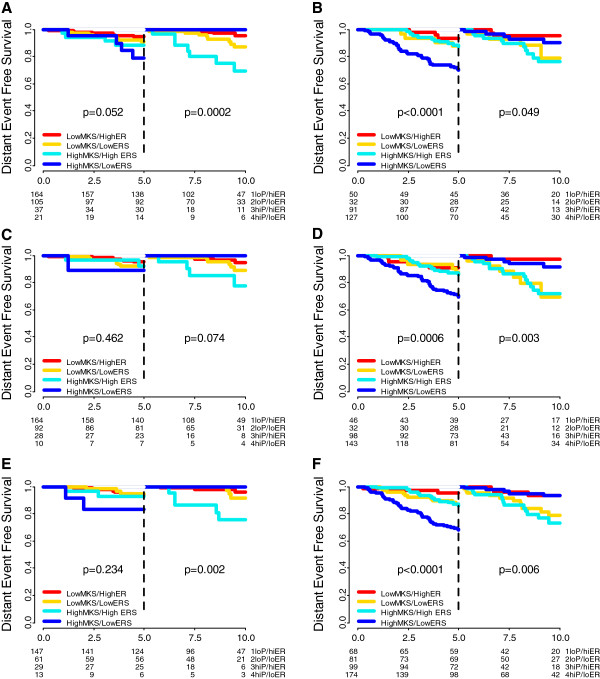
**Time-dependent assessment of combinations of different markers of proliferation and estrogen receptor (ER)-related genes.** Landmark analyses of the Kaplan-Meier estimates of distant event-free survival (DEFS) during the first 5 years (left side of each panel) and from 5 to 10 years (right side of each panel) are shown for tamoxifen-treated patients according to four biomarker groups by combining mitosis kinase score (MKS) (low and high) and estrogen-related score (ERS) (high and low) in ER-positive/human epidermal growth factor (HER)2-negative low proliferation tumors **(A)** and high-proliferation tumors **(B)** by the three-gene model [21]; in Luminal A **(C)** and luminal B **(D)** by PAM50; and in low risk **(E)** and high risk **(F)** by Mammaprint. loP, low MKS; hiP, high MKS; loER, low ERS; hiER, high ERS.

Furthermore, the four biomarker groups based on MKS and ERS could further risk-stratify patients for early (<5 years) and late relapses (within 5 to 10 years) within prognostic risk groups assigned by three first-generation genomic prognostic predictors, including the three-gene model (Figure 3A and 3B), luminal A and B molecular subtypes by PAM50 (Figure 3C and 3D) and low- and high-risk groups by Mammaprint (Figure 3E and 3F).

### Confirmation of results in an independent cohort of patients treated with adjuvant tamoxifen

**Figure 4:**
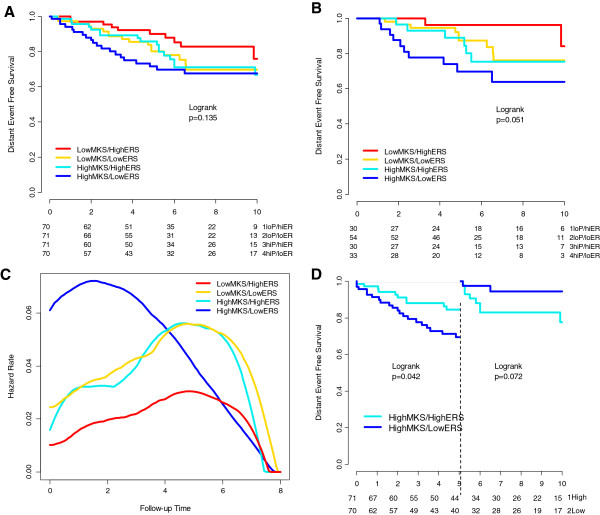
**Prognostic values of the four biomarker groups in the second cohort of tamoxifen-treated patients.** Kaplan-Meier estimates of 10-year distant event-free survival (DEFS) of the four biomarker groups obtained by combining low and high mitosis kinase score (MKS) and estrogen-related score (ERS) in all patients **(A)** and node-negative patients **(B)**. Annual hazard rates for distant recurrences by biomarker-group estimates using kernel-based methods for all patients **(C)**. Landmark analyses of the Kaplan-Meier estimates of DEFS during the first 5 years (left side of the panel) and from 5 to 10 years (right side of the panel) is shown for the high-MKS groups by ERS (high and low) **(D)**. loP, low MKS; hiP, high MKS; loER, low ERS; hiER, high ERS).

The patient characteristics of an independent series of ER-positive patients treated with adjuvant tamoxifen are described in Table 1. The median follow up was 7.2 years. The median cutoff point was applied to MKS and ERS. The time-varying pattern of distant relapse was qualitatively similar to that observed in the previously described cohort (Figure 4A-C). In the high-proliferation group the risk of relapse for the high-ERS group was lower in the first 5 years (*P* = 0.057) and higher after 5 years (*P* = 0.072) (Figure 4D). We also confirmed that in the first 2.5 years the majority of the relapses occurred in the high-MKS/low-ERS groups in node-negative but not in node-positive patients (Additional file 4: Figure S8).

### Predictive values of proliferation and ER-associated genes in tumors treated with neoadjuvant letrozole

**Figure 5:**
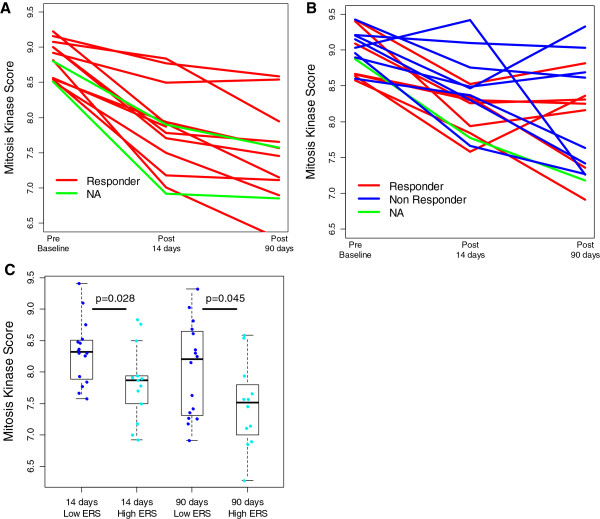
**Predictive value of the estrogen-related score (ERS) in the high-proliferation group after neoadjuvant letrozole.** The dynamic change of proliferation measured by mitosis kinase score (MKS) at baseline, 14 and 90 days in patients with baseline high proliferation treated with neoadjuvant letrozole in the high-ERS **(A)** and low-ERS **(B)** groups (red line = clinical response; blue line = non responder; green line = clinical information not available). **(C)** Box plot describing the proliferation at 14 and 90 days in the high-ERS and low-ERS groups. A comparison between MKS values in different groups was performed by Wilcoxon rank-sum test.

We evaluated the relationship between MKS and ERS and clinical and molecular response to neoadjuvant letrozole (n = 58). Molecular response was defined as downregulation of the MKS, as a measure of proliferation, at 14 and 90 days during neoadjuvant therapy. Among cancers with high proliferation (that is, high MKS) at baseline (n = 26), the clinical response rates were 47% (7/15) and 100% (11/11) for the low-ERS and high-ERS groups, respectively (*P* = 0.013). High-ERS cancers also had a more significant downregulation of proliferation at 14 (*P* = 0.028) and at 90 days (*P* = 0.045) compared to low-ERS tumors in response to letrozole therapy (Figure 5). These findings confirm lesser endocrine sensitivity of low-ERS cancers and are consistent with the observation of high early relapse rates among the high MKS/low ERS cancers.

## Discussion

The potential time-dependency of prognostic and predictive variables is well-known [11,24,25], but it is rarely studied purposefully. In this paper we examined the time-dependent interaction of proliferation [17] and ER-related gene expression [18,26] with the risk of early versus late recurrence in ER-positive early-stage breast cancer. We show that the high risk of relapse of highly proliferative tumors decreases over time in both untreated and tamoxifen-treated patients [11,24]. We also discovered a strong time-dependent prognostic interaction between the ERS and proliferation in tamoxifen-treated patients. The low risk of early recurrence in high-ERS, high-proliferation cancers during the first 5 years of active therapy converts into a higher risk of recurrence after 5 years. Highly proliferative cancers with strong ER signaling (high-MKS/high-ERS) show the greatest absolute increase for risk of late relapse after completion of 5 years of endocrine therapy, and therefore may be the best candidates for extended endocrine treatment. Among the low-proliferation cancers, the low-ERS group had constantly higher risk of relapse over the 10-year period compared to low-proliferation, high-ERS cancers. Extended endocrine therapy in this group also deserves further study. The time-dependent interaction between proliferation and estrogen signaling in predicting recurrence may explain why biomarkers that do not take into account such information (PAM50, Oncotype DX) do not predict late recurrences well [4,5].

The majority of the relapses within the first 2.5 years on tamoxifen occurred in the high-proliferation/low-ERS group. In a series of ER-positive patients treated with neoadjuvant letrozole, this group also had low clinical response rates and had minimal decrease in proliferation metrics [27]. These data suggest that the high-proliferation/low-ERS group is enriched in tumors intrinsically resistant to both tamoxifen and letrozole and in part explain why the use of upfront aromatase inhibitors (AIs) was not superior to the sequence of tamoxifen followed by an aromatase inhibitor in unselected patients [28,29]. Clinical trials could be designed and investigate specific strategies to overcome intrinsic resistance in these molecular groups. Additionally, node-negative and node-positive tumors show a different pattern of relapse in this early period, indeed almost all the recurrences in the molecular groups different from the high-proliferation/low-ERS group occur in node-positive cancers (*P* = 0.0008). The observation is consistent with findings in the BIG1-98 trial that showed a benefit from upfront letrozole compared to tamoxifen only in node-positive tumors during the short median follow up of 2.5 years [30].

Highly proliferative tumors relapsing after adjuvant tamoxifen had a progressively higher ERS score in late recurrences compared to early recurrences, but the same association was not observed in untreated tumors. One can speculate that early relapses are most common in tumors intrinsically resistant to endocrine treatment, while late recurrences occur also in tumors that could have relapsed earlier without treatment, but relapse was delayed due to adjuvant therapy. Our observation could also explain why in different reports the risk-reduction is modest around 20% for upfront AIs compared to tamoxifen [31], while it increases to 30% when tamoxifen is switched to an AI after 2 to 3 years [31], and the risk reduction is up to 50% when the AI is administered after 5 years of tamoxifen [1].

Our results are consistent with other reports and show that tumor size and nodal status remain independent prognostic factors. Clinical and molecular information, particularly nodal status should be used in combination for decision-making about extended endocrine treatments [32].

We acknowledge that the different characteristics of patients in the tamoxifen-treated and untreated cohorts call for caution in any comparison between the two groups. Also, our analysis is not based on data from randomized clinical trials but from prospectively assembled cohorts of cases for prognostic marker studies. The cohorts have been annotated and published in peer-reviewed journals [21], and the validity of clinical information has been demonstrated in several different studies [6,8,33]. The generalizability of our results was assessed in an independent cohort of tamoxifen-treated patients, and also supported by recently presented data by other groups [34,35].

## Conclusions

Our findings suggest that patients with highly proliferative (high MKS or high GGI) and high-ER breast cancers (high expression of ER-related genes both adopted from Oncotype DX or from PAM50) have the highest absolute risk for late relapse tumors, despite the fact that their risk of recurrence is modest in the first 5 years on tamoxifen. These tumors could be the best candidates for extended endocrine therapy. Patients with low proliferation and low-ER-related genes also remain at risk for late relapses, and benefit from extended endocrine treatment with aromatase inhibitors should be also assessed.

Early relapses (in the first 2.5 years) are highest in highly proliferative/low-ERS cancers, in particular in node-negative tumors, and this group also had minimal response to neoadjuvant letrozole. These findings suggest that this molecular group is enriched in tumors intrinsically resistant to endocrine therapy (both tamoxifen and letrozole) and in part can explain why upfront AIs were not superior to the sequence of tamoxifen followed by an AI in unselected patients.

Overall, the combined use of markers of proliferation and ER-related genes can inform the design of future adjuvant trials and assist in the selection of patients for extended endocrine treatment.

## Abbreviations

AI: Aromatase inhibitors; DEFS: Distant event-free survival; ER: Estrogen receptor; ERS: Estrogen-related score; GGI: Genomic grade index; HER2: Human epidermal growth factor receptor 2; HR: Hazard ratio; MKS: Mitosis kinase score; T: Tumor.

## Competing interests

W Fraser Symmans: Consultant or Advisory Role: Nuvera Biosciences (uncompensated); Stock Ownership: Nuvera Biosciences. C Sotiriou is named inventor on a patent application for the Gene expression Grade Index (GGI) used in this study. The other authors declare that they have no competing interests.

## Authors’ contributions

GB conceived and designed the study, performed the statistical analysis, contributed to the acquisition and collection of the gene expression data, data interpretation and writing the manuscript. LP and LG contributed to the study design, data analysis and interpretation and in writing the manuscript. TK contributed to the acquisition and collection of the gene expression data, statistical analysis, data interpretation and writing the manuscript. TI contributed to statistical analysis, data interpretation and writing the manuscript. YQ contributed to the statistical analysis and gene expression data normalization. AR, VM, MS, UH, SB, CS, BHK and WFS contributed to the acquisition and collection of the gene expression data, participated in data assembling and interpretation, and critically revised the manuscript. CMK, LS, AF, GDC and MZ participated in data assembling, analysis and interpretation and in writing the manuscript. All authors read and approved the final manuscript.

## Supplementary Material

Additional file 1: Table S1 Genes and probe sets used to define the mitosis kinase score (MKS), estrogen-related score (ERS) and luminal estrogen-related genes. Click here for file

Additional file 2 Supplementary methods. Click here for file

Additional file 3: Table S2 Univariate Cox analysis of clinical variables and molecular markers for untreated patients. Click here for file

Additional file 4: Figure S1 Time-dependent assessment of grade, genomic grade index (GGI) and PAM50 in untreated patients. **Figure S2.** Hazard rates for recurrences by biomarker groups in untreated and tamoxifen-treated patients. **Figure S3.** Time-dependent effect of estrogen-related score (ERS) by tertiles in high-mitosis kinase score (MKS) tumors (tamoxifen-treated patients). **Figure S4.** Prognostic values of biomarkers for early relapse in untreated and tamoxifen-treated patients. **Figure S5.** Baseline expression of ERS for tumor relapse in different time cohorts. **Figure S6.** Assessment of the time-dependent prognostic values of MKS, GGI and estrogen receptor (ER)-related luminal genes. **Figure S7.** Correlation between ESR1, ERS and luminal ER-related genes. **Figure S8.** Prognostic values of biomarkers for early relapse in tamoxifen-treated patients (cohort 2). Click here for file
