# Supplementary material for: Proliferation and estrogen signaling can distinguish patients at risk for early versus late relapse among estrogen receptor positive breast cancers
Source: Breast Cancer Res. 2013 Sep 23;15(5):R86. doi: 10.1186/bcr3481 (PMC3978752; doi:10.1186/bcr3481)
Supplement: Additional file 2 — Supplementary methods. [file bcr3481-S2.doc]

Supplementary Methods

*Tumor samples*

All of the gene expression profiles were generated with Affymetrix-based platforms (U133A: n=1251 and U133PLUS2.0: n=163). CEL file of unique breast cancer patients used in this study were retrieved from Gene Expression Omnibus (GEO at the web site http://www.ncbi.nlm.nih.gov/geo/).

To assess context-specific prognostic value (mixed prognostic/predictive value) of biomarkers in patients treated with adjuvant tamoxifen for 5 years, the following series were used: GSE6532 [1], GSE9195 [2], GSE17705 [3] and GSE12093 [4]. We also used as independent confirmatory cohort of our findings a series of ER-positive patients treated with adjuvant tamoxifen for 5 years (GSE26917) [5].

To assess pure prognostic value of biomarkers in node-negative, untreated patients, the following series were used: GSE2034 [6], GSE7390 [7], GSE11121 [8], GSE5327 [9], GSE2990 [10], and GSE6532 [1].

Duplicated patients were removed from the tamoxifen datasets (overlapping between GSE17705 and GSE6532 series) and untreated datasets (overlapping between GSE7390 and GSE2990). Because all these dataset are public available they have been used and described also by other investigators. In particular, an in deep description of the sample ID and clinical characteristics of patients included in this series can be found in the supplementary table (jnci-JNCI-11-0924-s02) of the manuscript from Haibe-Kains and co-authors [11]. Compared to the mentioned manuscript, we were not able to properly download two CEL files from GEO (GSM441874 and GSM151261), and we included 9 samples of the GSE6532 series ( 103B41, 147B19, 189B83, 193B72, 245C22, 46A25, 71A50, 76A44, 79A35) which were not previously reported because outcome information are not available for correlations between four marker groups and clinico-pathological variables.

To assess predictive value of biomarkers in ER-positive (Allred score 5 to 8) tumors treated with neoadjuvant endocrine-therapy (letrozole), we used the following series: GSE20181 [12]. In this series gene expression profiles for the same tumor at different time were available (at baseline, and at 14 and 90 days during treatment). We used in our analysis patients for which tumor assessment has been performed at least at baseline and at 14 days (58 patients) (for two patients the gene expression profile were not available at 90 days).

*Gene expression normalization*

MAS5 normalization was performed to a median target array intensity of 600 and data was transformed to log2 values using the Bioconductor software and Simpleaffy package ([www.bioconductor.org](http://www.bioconductor.org/)) as previously reported [13].  To make comparable results for the Affymetrix U133 Plus 2.0 with results generated using the U133A we convert CEL file for the U133Plus 2.0 for using only the probsets in common with the U133A before applying the normalization step. Probe sets were annotated using BRBArrayTools by the Bioconductor annotation package hgu133a.db (Version:2.4.1).

*Biomarker definition*

The proliferation score was calculated as the average expression of 12 mitotic kinases which have been previously reported and defined (Supplementary file 1, Table S1) [13]. The Estrogen-Related Score or ERS was defined as the average expression of the four genes adopted from the ER group form the Onco*type* DX [14]. Where more probe sets were available for one gene, the probe set with the highest average expression was selected (Supplementary file 1, Table S1). To assess the generalizability of results by combining “proliferation markers” and “estrogen-related gene expression markers” beyond the specific use of MKS and ERS, we will assess also the Genomic Grade Index as proliferation marker, and a luminal gene score adopted from PAM50 as estrogen-related genes marker. For this score [15], the probe set with the highest average expression has been selected for each of the seven genes represented on the affymetrix platform (Supplementary file 1 Table S1). Probe sets corresponding to the GPR160 gene are not available on the affymetrix U133a platform.

Median values for MKS, ERS and luminal scores were defined including patients treated with adjuvant-tamoxifen and untreated and four groups were created. Because of the small number of patients included in the neoadjuvant series, to avoid unbalance in the number of patients for each biomarker groups, the median values for MKS and ERS were re-defined on the 58 samples of this series.

The HER2 receptor status were defined according to three-gene model developed by Haibe-Kains and colleagues (ER, HER2 and AURKA) as reported in the supplementary table of their manuscript [11]. For the untreated series of patients, only ER-positive tumors were considered (defined as >10.18, log 2 value of the probe set “205225_at” as previously reported) [13].

The Genomic Grade Index risk groups, the Mammaprint classes and PAM50 molecular subtypes were defined as reported in the supplementary table (jnci-JNCI-11-0924-s02) of the manuscript from Haibe-Kains and co-authors [11]. The normal-like group defined according to the PAM50 classifier was not included in outcome analysis as also suggested by the group who originally develop the test [16].

References Supplementary Methods

1. Loi S, Haibe-Kains B, Desmedt C, Lallemand F, Tutt AM, Gillet C, Ellis P, Harris A, Bergh J, Foekens JA *et al*: **Definition of clinically distinct molecular subtypes in estrogen receptor-positive breast carcinomas through genomic grade**. *J Clin Oncol* 2007, **25**(10):1239-1246.

2. Loi S, Haibe-Kains B, Desmedt C, Wirapati P, Lallemand F, Tutt AM, Gillet C, Ellis P, Ryder K, Reid JF *et al*: **Predicting prognosis using molecular profiling in estrogen receptor-positive breast cancer treated with tamoxifen**. *BMC Genomics* 2008, **9**(239):239.

3. Symmans WF, Hatzis C, Sotiriou C, Andre F, Peintinger F, Regitnig P, Daxenbichler G, Desmedt C, Domont J, Marth C *et al*: **Genomic index of sensitivity to endocrine therapy for breast cancer**. *J Clin Oncol* 2010, **28**(27):4111-4119.

4. Zhang Y, Sieuwerts AM, McGreevy M, Casey G, Cufer T, Paradiso A, Harbeck N, Span PN, Hicks DG, Crowe J *et al*: **The 76-gene signature defines high-risk patients that benefit from adjuvant tamoxifen therapy**. *Breast Cancer Res Treat* 2009, **116**(2):303-309.

5. Filipits M, Rudas M, Jakesz R, Dubsky P, Fitzal F, Singer CF, Dietze O, Greil R, Jelen A, Sevelda P *et al*: **A new molecular predictor of distant recurrence in ER-positive, HER2-negative breast cancer adds independent information to conventional clinical risk factors**. *Clin Cancer Res* 2011, **17**(18):6012-6020.

6. Wang Y, Klijn JG, Zhang Y, Sieuwerts AM, Look MP, Yang F, Talantov D, Timmermans M, Meijer-van Gelder ME, Yu J *et al*: **Gene-expression profiles to predict distant metastasis of lymph-node-negative primary breast cancer**. *Lancet* 2005, **365**(9460):671-679.

7. Desmedt C, Piette F, Loi S, Wang Y, Lallemand F, Haibe-Kains B, Viale G, Delorenzi M, Zhang Y, d'Assignies MS *et al*: **Strong time dependence of the 76-gene prognostic signature for node-negative breast cancer patients in the TRANSBIG multicenter independent validation series**. *Clin Cancer Res* 2007, **13**(11):3207-3214.

8. Schmidt M, Bohm D, von Torne C, Steiner E, Puhl A, Pilch H, Lehr HA, Hengstler JG, Kolbl H, Gehrmann M: **The humoral immune system has a key prognostic impact in node-negative breast cancer**. *Cancer Res* 2008, **68**(13):5405-5413.

9. Minn AJ, Gupta GP, Padua D, Bos P, Nguyen DX, Nuyten D, Kreike B, Zhang Y, Wang Y, Ishwaran H *et al*: **Lung metastasis genes couple breast tumor size and metastatic spread**. *Proc Natl Acad Sci U S A* 2007, **104**(16):6740-6745.

10. Sotiriou C, Wirapati P, Loi S, Harris A, Fox S, Smeds J, Nordgren H, Farmer P, Praz V, Haibe-Kains B *et al*: **Gene expression profiling in breast cancer: understanding the molecular basis of histologic grade to improve prognosis**. *J Natl Cancer Inst* 2006, **98**(4):262-272.

11. Haibe-Kains B, Desmedt C, Loi S, Culhane AC, Bontempi G, Quackenbush J, Sotiriou C: **A three-gene model to robustly identify breast cancer molecular subtypes**. *J Natl Cancer Inst* 2012, **104**(4):311-325.

12. Miller WR, Larionov A, Anderson TJ, Evans DB, Dixon JM: **Sequential changes in gene expression profiles in breast cancers during treatment with the aromatase inhibitor, letrozole**. *Pharmacogenomics J* 2012, **12**(1):10-21.

13. Bianchini G, Iwamoto T, Qi Y, Coutant C, Shiang CY, Wang B, Santarpia L, Valero V, Hortobagyi GN, Symmans WF *et al*: **Prognostic and therapeutic implications of distinct kinase expression patterns in different subtypes of breast cancer**. *Cancer Res* 2010, **70**(21):8852-8862.

14. Paik S, Shak S, Tang G, Kim C, Baker J, Cronin M, Baehner FL, Walker MG, Watson D, Park T *et al*: **A multigene assay to predict recurrence of tamoxifen-treated, node-negative breast cancer**. *N Engl J Med* 2004, **351**(27):2817-2826.

15. Parker JS, Mullins M, Cheang MC, Leung S, Voduc D, Vickery T, Davies S, Fauron C, He X, Hu Z *et al*: **Supervised risk predictor of breast cancer based on intrinsic subtypes**. *J Clin Oncol* 2009, **27**(8):1160-1167.

16. Nielsen TO, Parker JS, Leung S, Voduc D, Ebbert M, Vickery T, Davies SR, Snider J, Stijleman IJ, Reed J *et al*: **A comparison of PAM50 intrinsic subtyping with immunohistochemistry and clinical prognostic factors in tamoxifen-treated estrogen receptor-positive breast cancer**. *Clin Cancer Res* 2010, **16**(21):5222-5232.
