# Supplementary material for: Proliferation and estrogen signaling can distinguish patients at risk for early versus late relapse among estrogen receptor positive breast cancers
Source: Breast Cancer Res. 2013 Sep 23;15(5):R86. doi: 10.1186/bcr3481 (PMC3978752; doi:10.1186/bcr3481)
Supplement: Additional file 4: Figure S1 — Time-dependent assessment of grade, genomic grade index (GGI) and PAM50 in untreated patients. Figure S2. Hazard rates for recurrences by biomarker groups in untreated and tamoxifen-treated patients. Figure S3. Time-dependent effect of estrogen-related score (ERS) by tertiles in high-mitosis kinase score (MKS) tumors (tamoxifen-treated patients). Figure S4. Prognostic values of biomarkers for early relapse in untreated and tamoxifen-treated patients. Figure S5. Baseline expression of ERS for tumor relapse in different time cohorts. Figure S6. Assessment of the time-dependent prognostic values of MKS, GGI and estrogen receptor (ER)-related luminal genes. Figure S7. Correlation between ESR1, ERS and luminal ER-related genes. Figure S8. Prognostic values of biomarkers for early relapse in tamoxifen-treated patients (cohort 2). [file bcr3481-S4.ppt]

## Slide 1
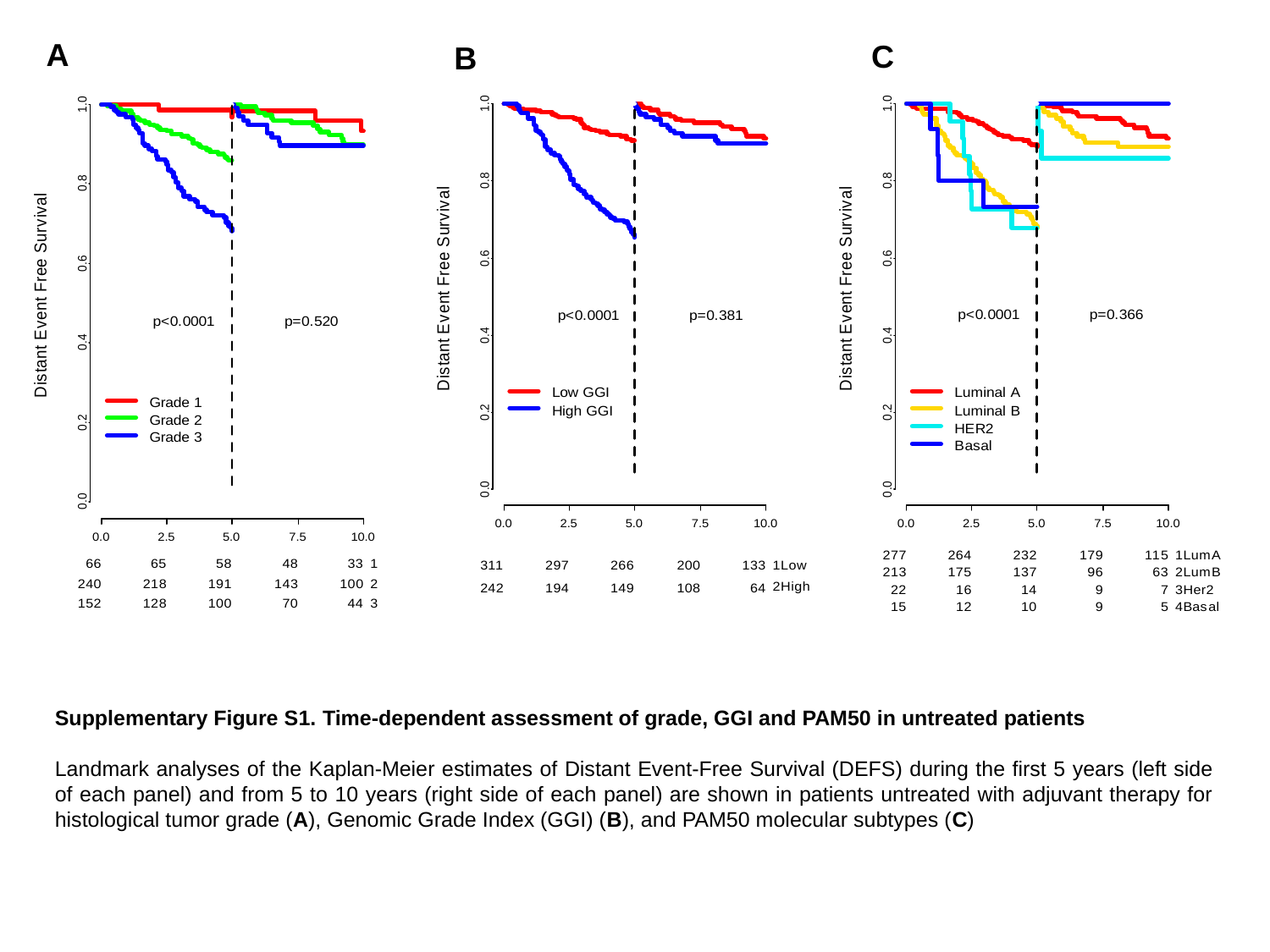

A
C
B
Supplementary Figure S1. Time-dependent assessment of grade, GGI and PAM50 in untreated patients
Landmark analyses of the Kaplan-Meier estimates of Distant Event-Free Survival (DEFS) during the first 5 years (left side of each panel) and from 5 to 10 years (right side of each panel) are shown in patients untreated with adjuvant therapy for histological tumor grade (A), Genomic Grade Index (GGI) (B), and PAM50 molecular subtypes (C)

## Slide 2
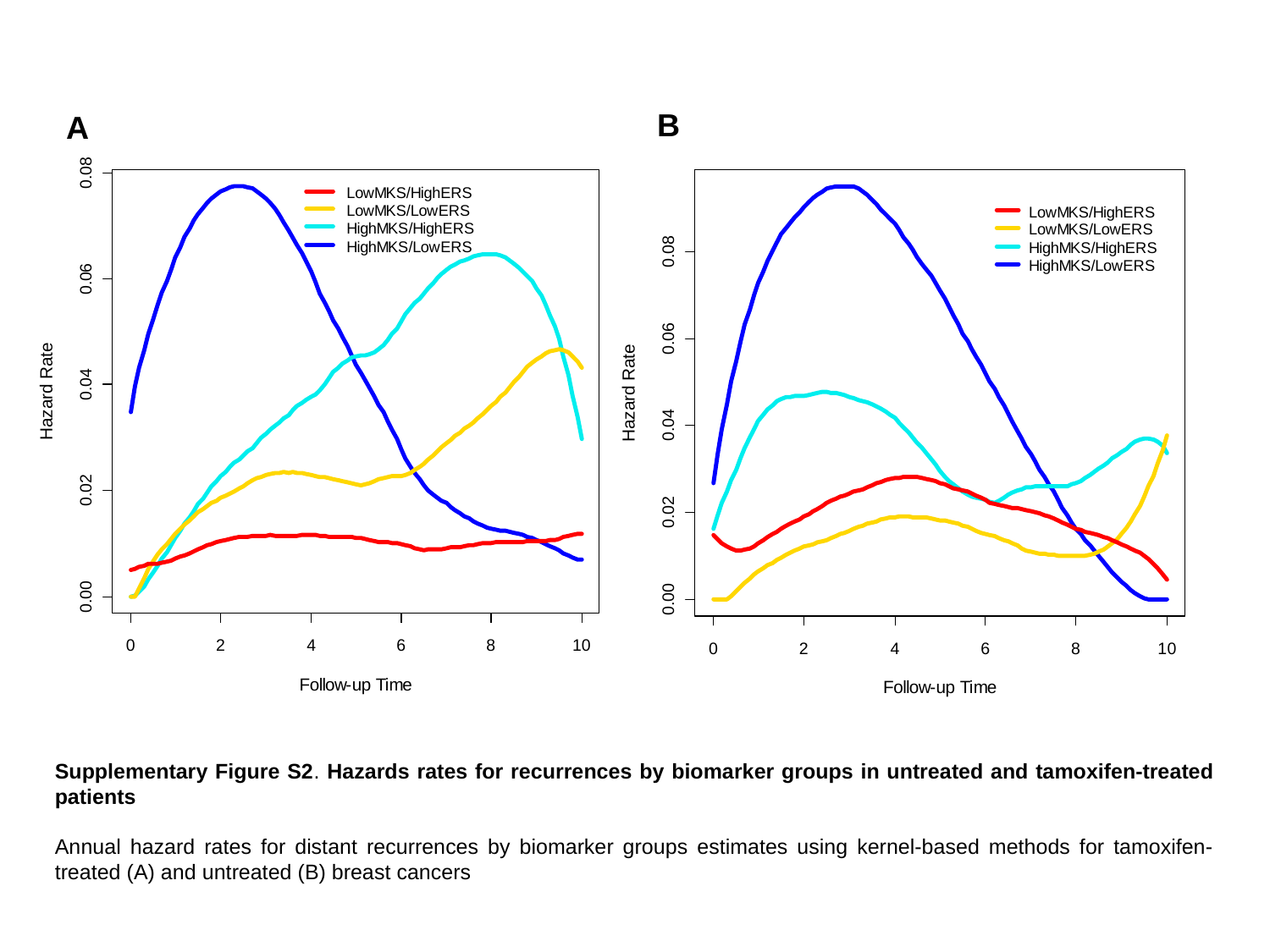

B
A
Supplementary Figure S2. Hazards rates for recurrences by biomarker groups in untreated and tamoxifen-treated patients
Annual hazard rates for distant recurrences by biomarker groups estimates using kernel-based methods for tamoxifen-treated (A) and untreated (B) breast cancers

## Slide 3
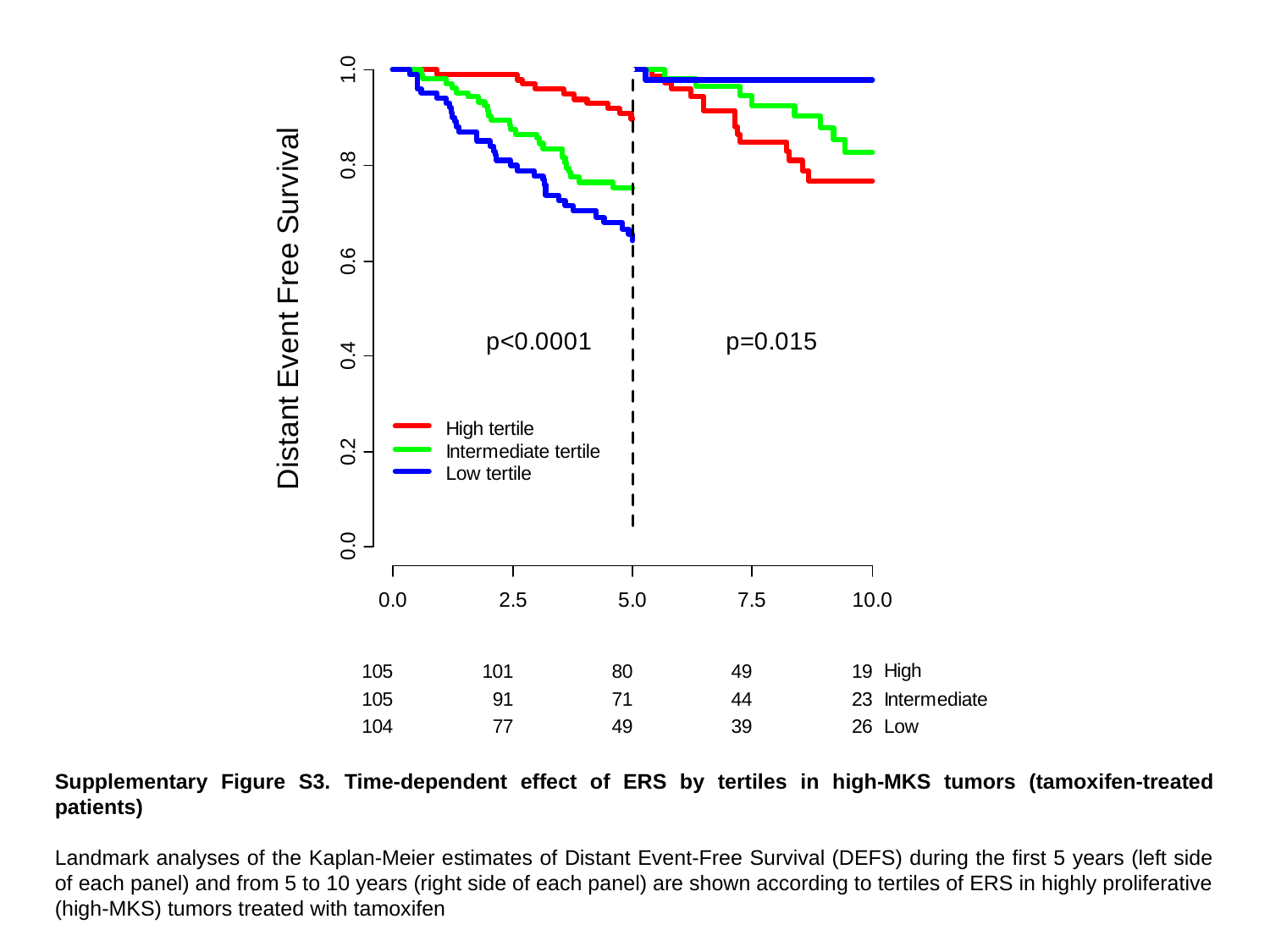

Supplementary Figure S3. Time-dependent effect of ERS by tertiles in high-MKS tumors (tamoxifen-treated patients)
Landmark analyses of the Kaplan-Meier estimates of Distant Event-Free Survival (DEFS) during the first 5 years (left side of each panel) and from 5 to 10 years (right side of each panel) are shown according to tertiles of ERS in highly proliferative (high-MKS) tumors treated with tamoxifen

## Slide 4
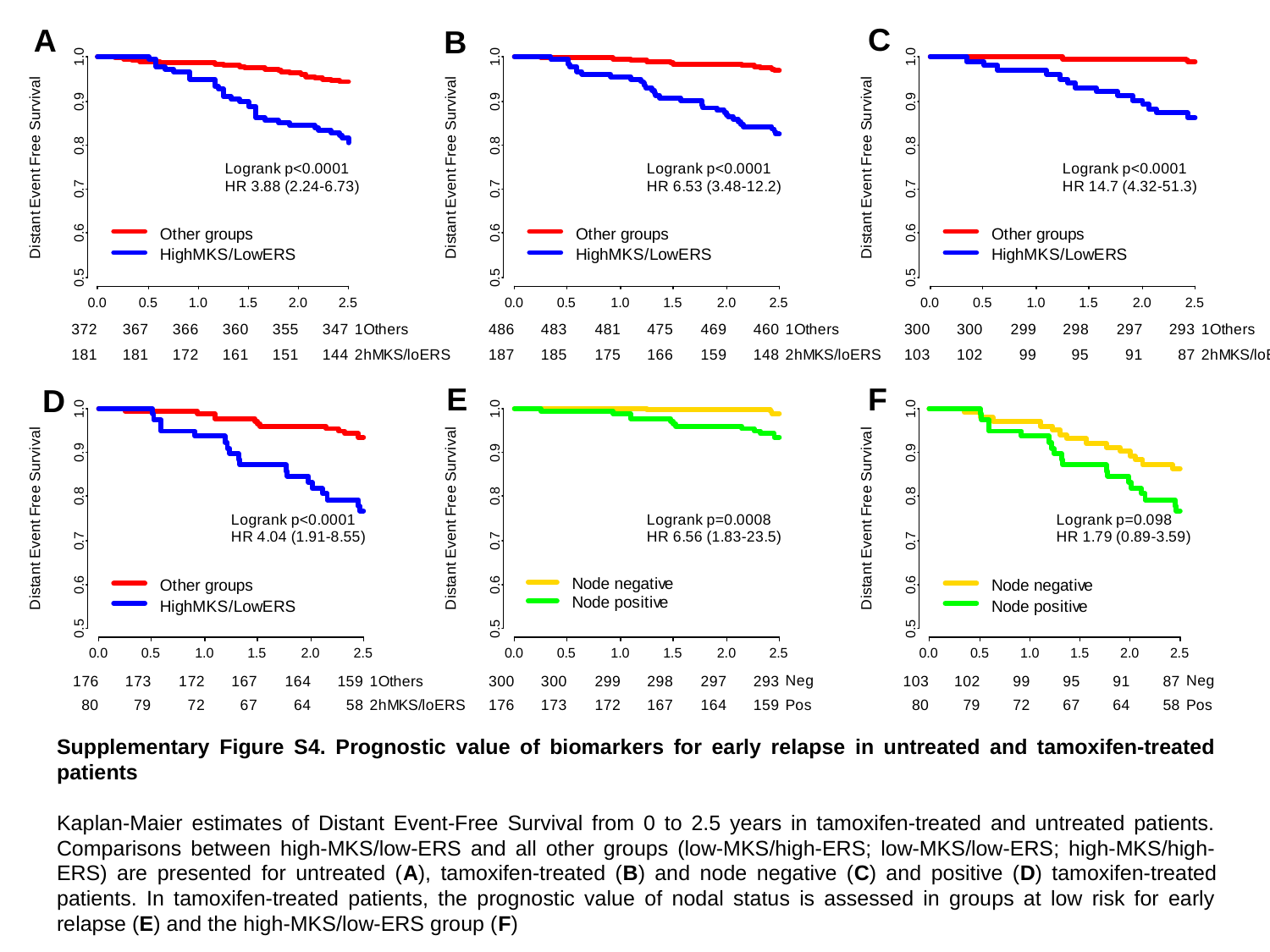

C
A
B
E
F
D
Supplementary Figure S4. Prognostic value of biomarkers for early relapse in untreated and tamoxifen-treated patients
Kaplan-Maier estimates of Distant Event-Free Survival from 0 to 2.5 years in tamoxifen-treated and untreated patients. Comparisons between high-MKS/low-ERS and all other groups (low-MKS/high-ERS; low-MKS/low-ERS; high-MKS/high-ERS) are presented for untreated (A), tamoxifen-treated (B) and node negative (C) and positive (D) tamoxifen-treated patients. In tamoxifen-treated patients, the prognostic value of nodal status is assessed in groups at low risk for early relapse (E) and the high-MKS/low-ERS group (F)

## Slide 5
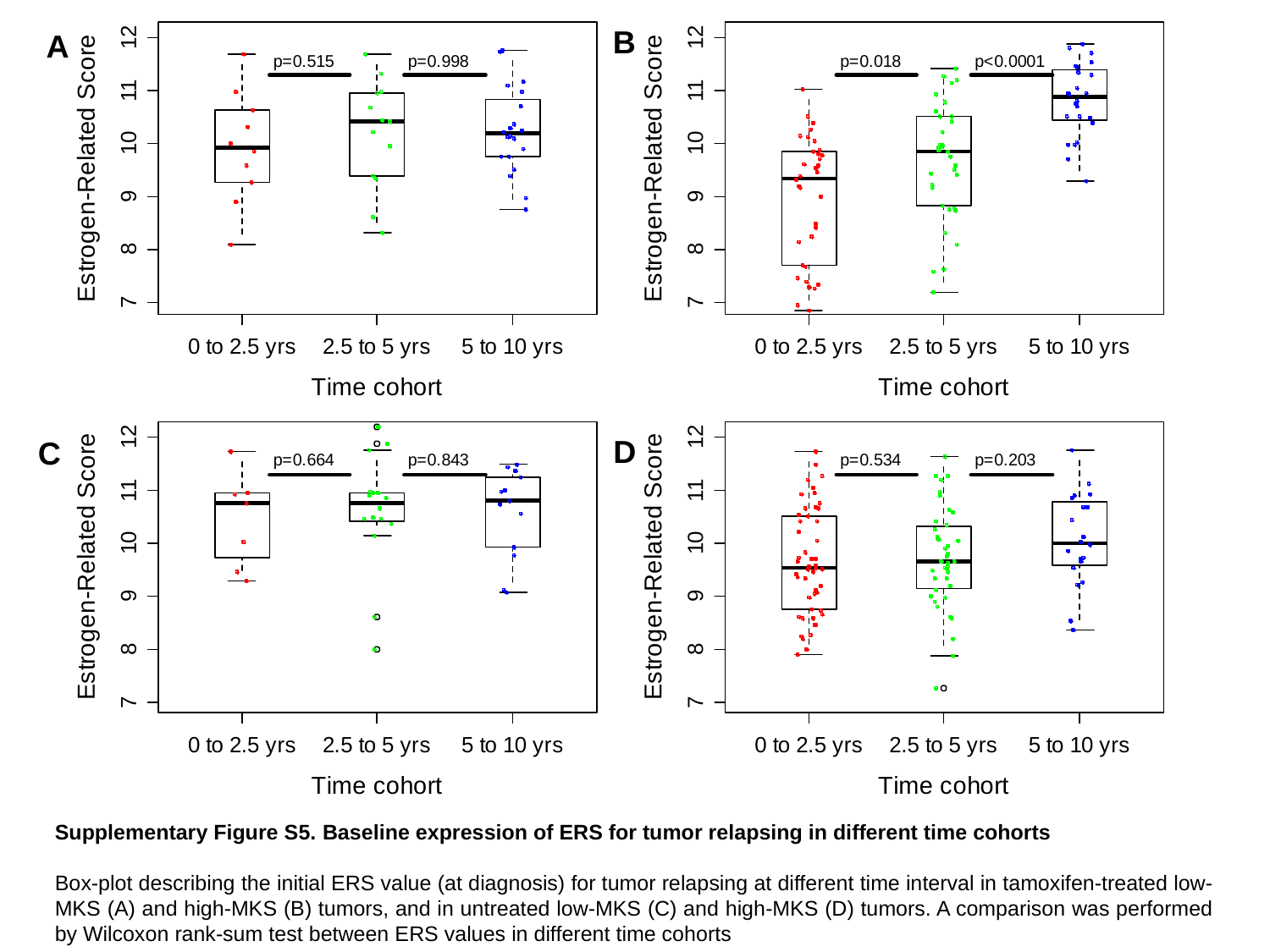

B
A
D
C
Supplementary Figure S5. Baseline expression of ERS for tumor relapsing in different time cohorts
Box-plot describing the initial ERS value (at diagnosis) for tumor relapsing at different time interval in tamoxifen-treated low-MKS (A) and high-MKS (B) tumors, and in untreated low-MKS (C) and high-MKS (D) tumors. A comparison was performed by Wilcoxon rank-sum test between ERS values in different time cohorts

## Slide 6
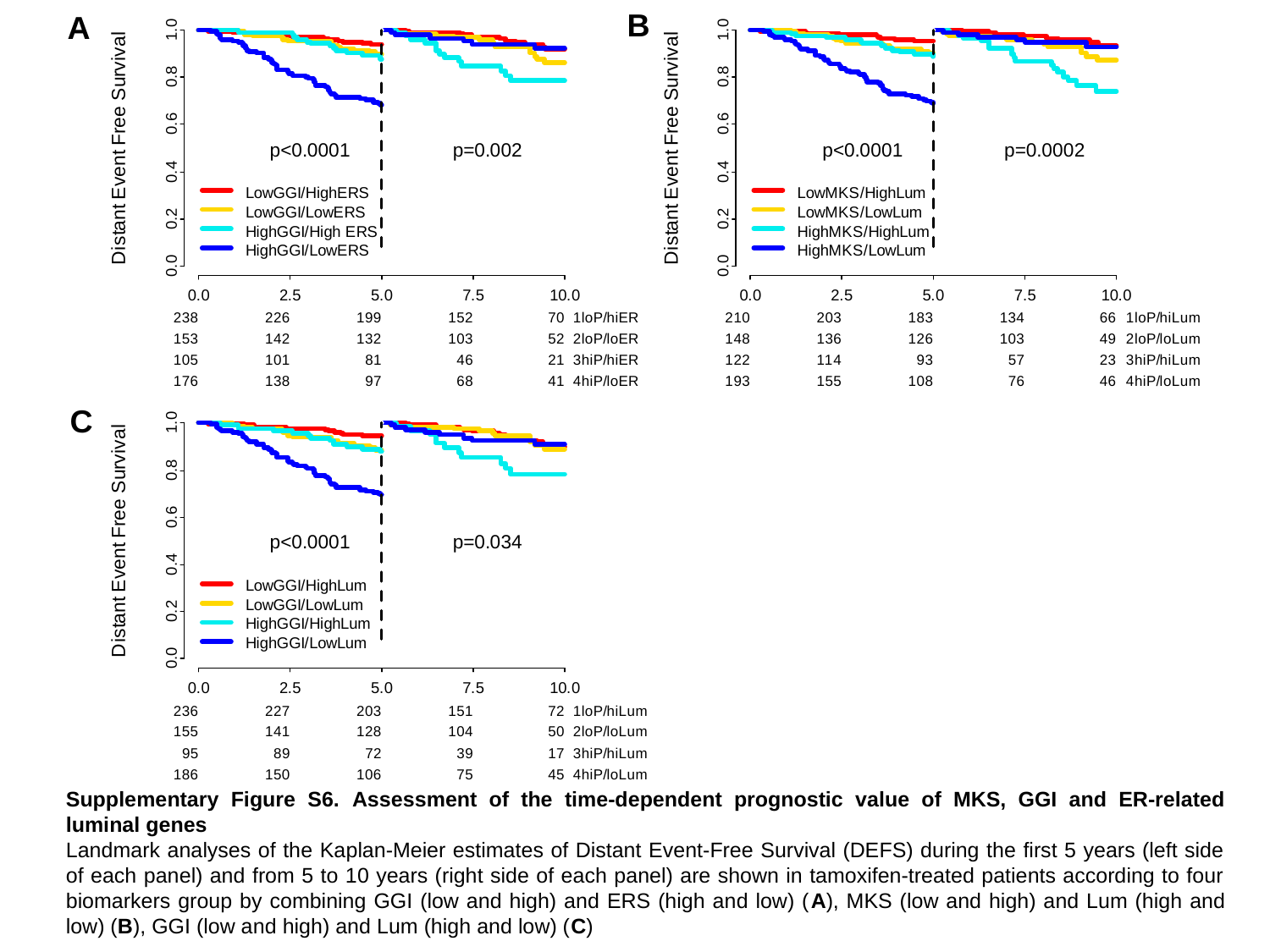

B
A
C
Supplementary Figure S6. Assessment of the time-dependent prognostic value of MKS, GGI and ER-related luminal genes
Landmark analyses of the Kaplan-Meier estimates of Distant Event-Free Survival (DEFS) during the first 5 years (left side of each panel) and from 5 to 10 years (right side of each panel) are shown in tamoxifen-treated patients according to four biomarkers group by combining GGI (low and high) and ERS (high and low) (A), MKS (low and high) and Lum (high and low) (B), GGI (low and high) and Lum (high and low) (C)

## Slide 7
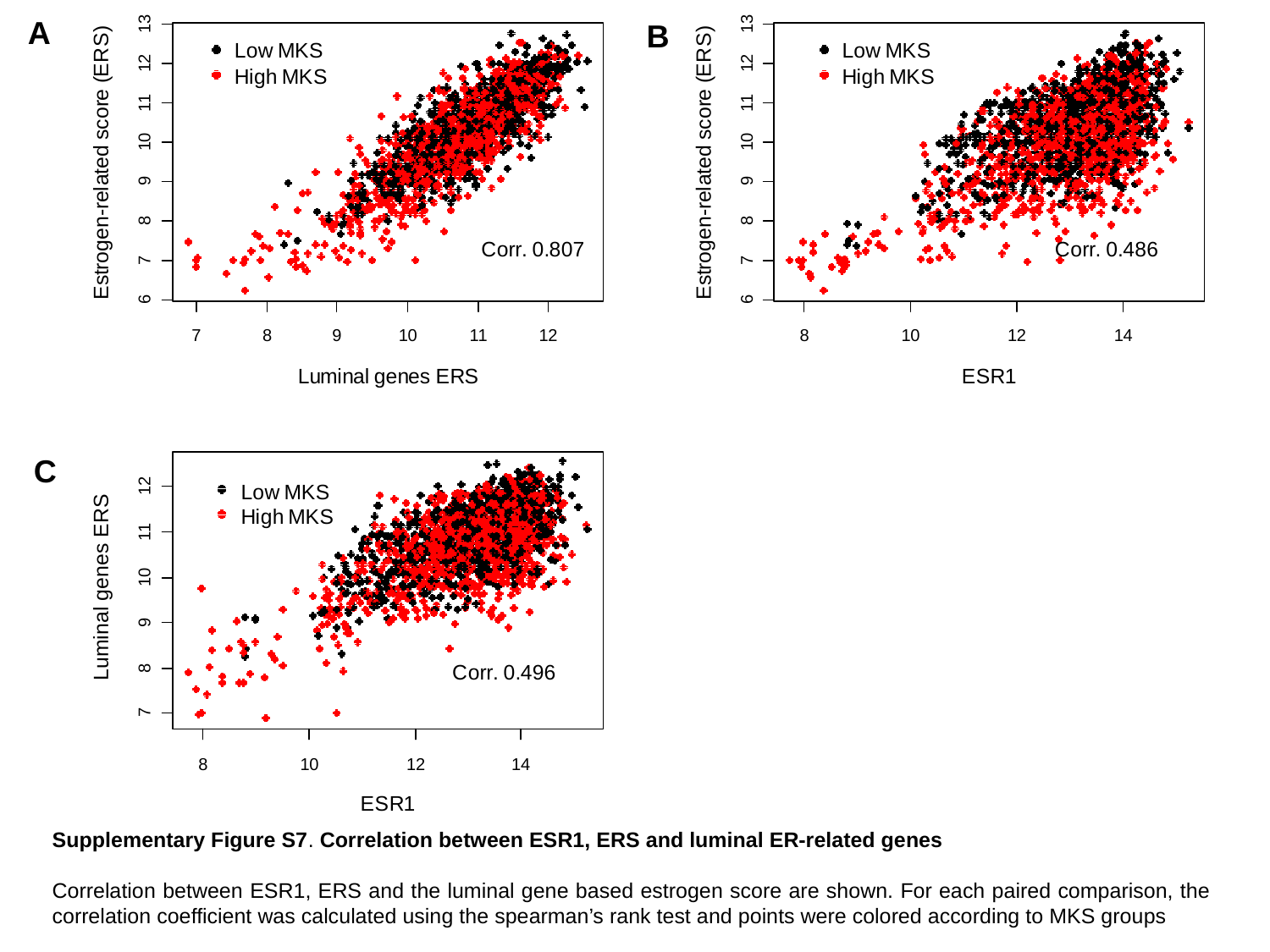

A
B
C
Supplementary Figure S7. Correlation between ESR1, ERS and luminal ER-related genes
Correlation between ESR1, ERS and the luminal gene based estrogen score are shown. For each paired comparison, the correlation coefficient was calculated using the spearman’s rank test and points were colored according to MKS groups

## Slide 8
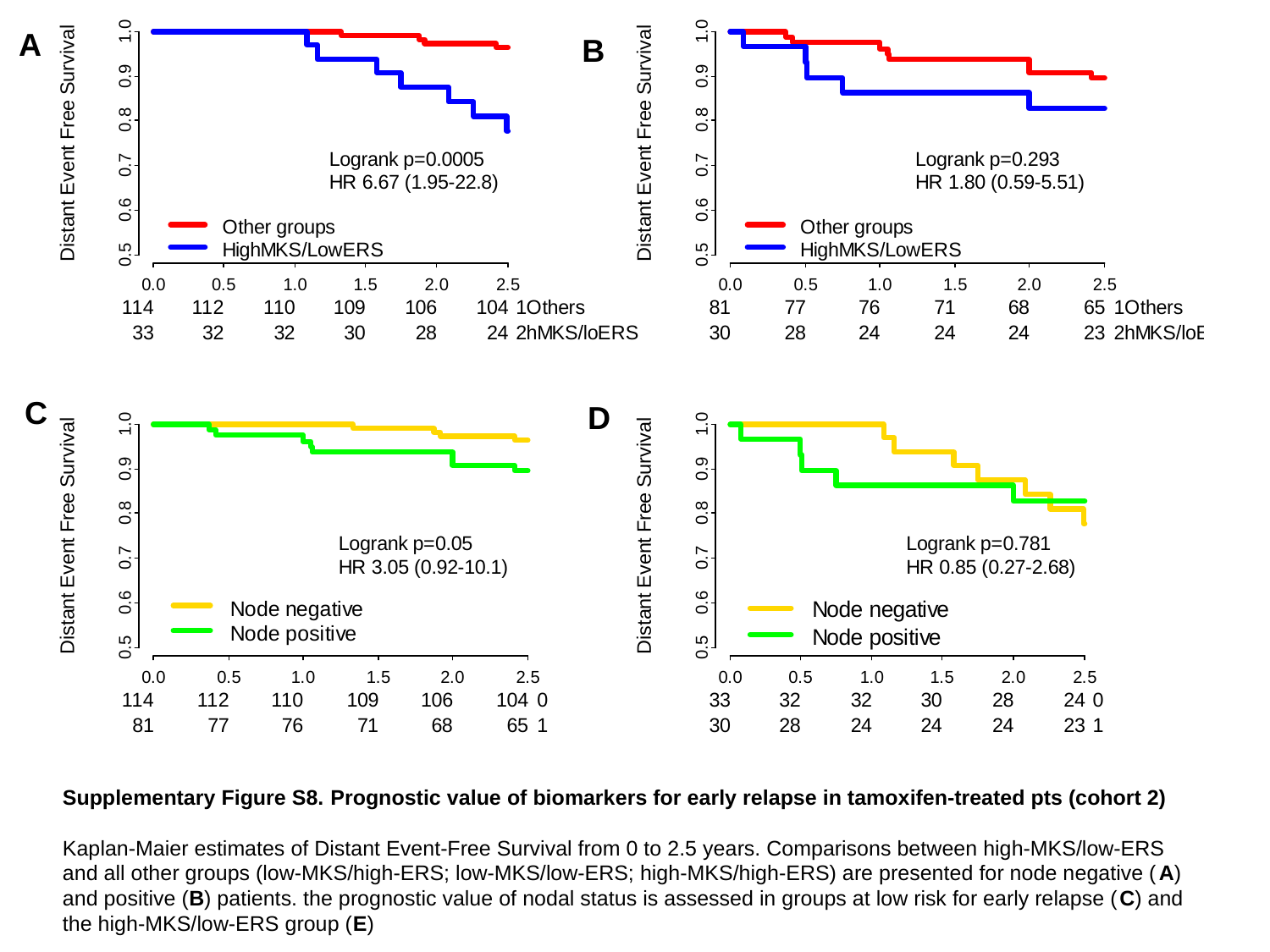

A
B
C
D
Supplementary Figure S8. Prognostic value of biomarkers for early relapse in tamoxifen-treated pts (cohort 2)
Kaplan-Maier estimates of Distant Event-Free Survival from 0 to 2.5 years. Comparisons between high-MKS/low-ERS and all other groups (low-MKS/high-ERS; low-MKS/low-ERS; high-MKS/high-ERS) are presented for node negative (A) and positive (B) patients. the prognostic value of nodal status is assessed in groups at low risk for early relapse (C) and the high-MKS/low-ERS group (E)
